# First generation migrants’ experiences of terminal illness: a systematic review of diasporic dying

**DOI:** 10.1186/s12904-025-01789-0

**Published:** 2025-05-26

**Authors:** Tim Sedgley, Joanne Alexander, Liz Forbat

**Affiliations:** University of Stirling, Stirling, FK9 4LA UK

**Keywords:** Migrants, Terminal, Palliative, Equality, Systematic review

## Abstract

**Background:**

Migration is an established global phenomenon. While many newly arrived migrants have better health than the general population of the country they have moved to, migrants also have their own healthcare needs and face particular issues when diagnosed with a terminal illness. First generation migrants are less likely to have social, financial, and medical supports when faced with a terminal illness. These factors make first generation migrants an important group to understand in order to inform service commissioning and delivery.

**Methods:**

The systematic review was an international qualitative evidence synthesis of English language papers from 2000 to 2023. The primary research question underpinning this novel review was: What are the experiences of first-generation migrants who live with or who are supporting a relative with a terminal illness in the country to which they have moved? Databases (MEDLINE; CINAHL; PsycINFO; SocIndex; Web of Science) were searched in August 2023. Records of 1593 publications were screened, resulting in 39 included papers. CASP was used to inform quality appraisal.

**Results:**

First generation migrants struggled with accessing suitable health services and treatments. Structural barriers, such as lack of support for translation/interpreting and for navigating care was visible alongside limited social support networks. Financial precarity ran as a thread through the data, with participants needing to work while unwell, and being unable to return to their country of origin for their own death or to bear witness to the deaths of relatives. First generation migrants experienced caregiving through the lens of difference; maintaining autonomy in the country they would die in, intersected with cultural practices and expectations such as not sharing the prognosis, and mis-matched ideas regarding quality of care provided. The identity of ‘migrant’ is heterogenous, poorly defined, and may have resulted in identifying studies conducted in the global north.

**Conclusions:**

Diasporic dying is not a new phenomenon, yet services and policies fail to meet people’s needs. Services urgently need to identify and dismantle structures which uphold and perpetuate inequality, including this population who suffer multiple disadvantages and risks.

**Protocol registration:**

CRD42023457054.

## Background

Many migrants form a core part of the health service workforce in the global north [1–4]. While many newly arrived migrants on work/study visas have better health than the general population of the country they have moved to [5–7], migrants also have their own healthcare needs, and face particular issues when diagnosed with a terminal illness. Diasporic dying, that is, the dying/death of people separated from their homelands, has yet to be fully understood.

Despite health and social care being basic human rights [8] and a key component of migrant integration [9], migrants can face limitations to the health care they access, and high costs [10]. For people with valid visas, what is free and what requires payment will vary country-by-country and by visa type. Undocumented migrants (those without a valid visa) encounter even greater challenges in accessing healthcare with restrictions on accessing hospice settings and medicines [11] where they are likely to have very limited access to healthcare. Research on marginalised communities affected by terminal illness identifies lower levels of service use including engagement with hospices [12, 13], but higher levels of need because they do not have local family carers [14]. The literature calls for more thoughtful culturally responsive approaches to service provision [15–18].

Migrants may also lack an intergenerational safety net and social capital of an extended family in the same country [19], meaning that terminal illness is experienced with limited local support which impairs coping and limits quality of life [20]. Migration is a major life experience, and when coupled with the further turmoil of a terminal illness, people in this situation face uniquely difficult circumstances [21].

Terminal illness also brings concerns beyond health. Poverty and deprivation have been invisible yet powerful influences over people’s experiences of dying [22–26]. In the UK alone, 90,000 people die in poverty each year [27]. This risk is particularly acute for those from Black and minoritized ethnic groups [28], with two fifths of working age people from minority ethnic backgrounds dying below the poverty line [27]. Destitution even for those without a life-shortening condition is also very high, with a fifth of destitute people in the UK being migrants [29], and with the UK seeing a 136% increase in destitute migrants since 2019 [30]. Consequently, the risks to migrants of poverty at end of life are substantial.

The complexities of migration, health, finances, and management of meaning-making are themes threaded through the lives and deaths of transnational people [31]. These experiences have been disseminated as poems and drawings depicting experiences of e.g. racism, history, politics, and heterogenous representations of belonging [32].

We were not able to identify any systematic reviews examining migrant experiences of terminal illness, save for one focused on terminally ill children who are forced migrants [33] and some scoping reviews on advance care planning [34], access to care [35] and older adults [18, 36]. Drawing together the literature on first generation migrants who are less likely to have social, financial, and medical supports offers the opportunity to fully understand the evidence and impacts identified within the literature. The driver for conducting this systematic review was from a prior (unrelated) qualitative study on working age people with a terminal illness. One participant was a migrant and described the profound impact being subject to a visa had on him, and how this framed his experience of terminal illness. Our motivation to conduct this review was to produce insights which could be used by services and commissioners in serving this marginalised group of dying diaspora.

The primary research question underpinning this review was: What are the experiences of first-generation migrants who live with or who are supporting a relative with a terminal illness in the country to which they have moved?

## Methodology

Thematic synthesis [37], an interpretative form of qualitative evidence synthesis, was selected as the systematic review methodology as this approach facilitates understanding of individuals’ and groups’ nuanced experiences and views [38].

The study was conducted with reference to Preferred Reporting Items for Systematic Reviews and Meta Analyses (PRISMA) reporting guidelines [39]. The protocol was registered on the International Prospective Register of Systematic Reviews (PROSPERO) reference: CRD42023457054.

### Eligibility

Studies were eligible for inclusion if the sample constituted:

1. First generation adult migrants with a terminal illness or significant other.
2. Relatives of a first-generation migrant who had a terminal illness and for whom they provided support.
3. Qualitative peer reviewed studies, including theses, and mixed methods studies, where qualitative data were reported separately and/or could easily be extracted, and which reported qualitative data related to the phenomenon of interest. Systematic reviews were also included if they reported third order analysis or quotations.
4. Published in English, between 2000 and 2023, from any country.

The date range 2000–2023 was chosen to capture a more contemporary picture of the research phenomena, and to ensure that data were also captured following the advent of the “hostile” immigration environment, which in the UK started in 2010, and culminated in the Nationality and Borders Act 2022 coming into effect. Studies published in any country were included to facilitate the potential to gain a deeper understanding of the phenomena of interest and any similarities and differences between countries and cultures. However, due to lack of foreign language fluency, and resources and time constraints, which precluded accurate and timely translation, studies not published in English were not included to maintain consistency in data extraction and quality appraisal. Preliminary scoping searches conducted suggest that relevant studies published in other languages were unlikely to significantly impact the overall findings and conclusions of the review.

Grey literature and reports were not included as they were unlikely to provide additional insights not included in peer review publications [40], where indexing lacks rigour to provide reassurance of identifying suitable sources [41, 42], and quality may be suboptimal [43].

Determining *first generation* status was not always easy as this was often not made explicit within the literature; proxies such as ‘interview conducted in first language’ were not used. Some populations did not fit within colonial and minority world labelling of ‘first generation’, for example studies with continually migratory populations such as gypsy/Roma/travellers. Such groups may not have had one specific point of migration, whether temporal, physical, or geographical, but are typically not described as ‘first generation migrants’.

The lack of explicitness may be due to lack of clarity and/or differences in the *migrant* definition. For example, in the UK there is no single legal definition of *migrant.* The term, when used in government data sources, such as the United Nations, Annual Population Survey, Office for National Statistics, Labour Force Survey, the Census, and Home Office visa data, is defined in different ways [44]. The opacity of the word migrant may account for the lack of clarity or absence of *migrant* definition in much of the literature, thus making the determination of first-generation status problematic.

### Search strategy and information sources

The SPiDER framework (Sample, Phenomenon of Interest, Design, Evaluation, and Research) was used [45] to define the critical components of the research question, establish inclusion criteria, and inform and standardise the search strategy (Table 1).

**Table 1:** Exemplar search strategy

S: Migrants on UK work-dependent visas	“transients and migrants”[Mesh] OR “emigration and immigration”[Mesh] OR “refugees” [Mesh] OR “foreign professional personnel” [Mesh] OR “ethnic groups” [Mesh] OR “emigrants and immigrants” OR “human migration” OR “foreign*” OR “foreign national*” OR “alien*” OR “non-native*” OR “non native*” OR “settler*” OR “newcomer*” OR “immigrant worker*” OR “migrant worker*” OR “migra*” OR “alien worker*” OR “foreign worker*” OR “non-native worker” OR “non native worker” OR “legal alien*” OR “immigra*” OR “emigra*” OR “first-generation” OR “first generation” OR “expat*” OR “asylum seekers” OR “nationalit*”
Pi: Life-limiting diagnosis	“critical illness” [Mesh] OR “terminally ill patients” [Mesh] OR “palliative care” [Mesh] OR “terminal care” [Mesh] OR “hospice care” [Mesh] OR “advance directives” [Mesh] OR “palliative care nursing” [Mesh] OR “palliative medicine” [Mesh] OR “palliative care” OR “life-limiting illness” OR “life limiting illness*” OR “life-limiting condition” OR “life limiting condition*” OR “life-limiting disease” OR “life limiting disease*” OR “life-limiting syndrome” OR “life limiting syndrome” OR “life-limiting disability” OR “life limiting disabilit*” OR “ life threatening disease” OR “life-threatening disease*” OR “life threatening condition” OR “life-threatening condition” OR “end of life” OR “end-of-life” OR “terminal condition*” OR “terminal disease*” OR “terminal illness” OR “terminal disability” OR “incurable illness” OR “incurable condition*” OR “incurable disease*” OR “end-stage illness*” OR “end-stage condition*” OR “end-stage disease*” OR “terminal” OR “hospice” OR “end of life” OR “end-of-life” OR “last year of life”
E: Lived experience	“life experiences” “critical illness”[Mesh] OR “terminally ill patients”[Mesh] OR “palliative care”[Mesh] OR “terminal care”[Mesh] OR “hospice care”[Mesh] OR “advance directives”[Mesh] OR “palliative care nursing”[Mesh] OR “palliative medicine”[Mesh] OR “bereavement”[Mesh] OR “attitude”[Mesh] OR “perception” [Mesh] OR “information needs”[Mesh] OR “life change events”[Mesh] OR “health” [Mesh] OR “employment”[Mesh] OR “work experiences”[Mesh] OR “employee rights”[Mesh] OR “right to health”[Mesh] OR “occupations and professions”[Mesh] OR “absenteeism”[Mesh] OR “work-life balance”[Mesh] OR “quality of life”[Mesh] OR “wellness”[Mesh] OR “reflection” [Mesh] OR “attitude to death” [Mesh] OR “attitude to health”[Mesh] OR “attitude to illness”[Mesh] OR “attitude to medical treatment”[Mesh] OR “financial stress”[Mesh] OR “financial losses”[Mesh] OR “debt, financial”[Mesh] OR “income”[Mesh] OR “poverty” [Mesh] OR “anxiety”[Mesh] OR “stress, psychological”[Mesh] OR “decision making”[Mesh] OR “adaptation, psychological“[Mesh] OR “coping”[Mesh] OR “defense mechanisms”[Mesh] OR “psychology, occupational”[Mesh] OR “emotions”[Mesh] OR “stress, occupational” [Mesh] OR “mental health”[Mesh] OR “legislation” [Mesh] OR “social dominance” [Mesh] OR “power”[Mesh] OR “government”[Mesh] OR “confidence”[Mesh] OR “trust”[Mesh] OR “communication barriers”[Mesh] OR “social capital”[Mesh] OR “health services accessibility”[Mesh] OR “social problems”[Mesh] OR “social deprivation”[Mesh] OR “intersectionality” [Mesh] OR “social behavior”[Mesh] OR “behavioral symptoms”[Mesh] OR “social identity”[Mesh] OR “social integration”[Mesh] OR “social inclusion”[Mesh] OR “social justice”[Mesh] OR “social welfare”[Mesh] OR “social perception”[Mesh] OR “socioeconomic disparities in health”[Mesh] OR “family”[Mesh] OR “spouses”[Mesh] OR “significant other”[Mesh] OR “caregivers”[Mesh] OR “extended family”[Mesh] OR “child”[Mesh] OR “cultural competence” [Mesh] OR “discrimination”[Mesh] OR “cultural sensitivity”[Mesh] OR “cultural diversity”[Mesh] OR “racial equality”[Mesh] OR “antiracism” [Mesh] OR “racism”[Mesh] OR “systemic racism”[Mesh] OR “palliative care” OR “life-limiting illness” OR “life limiting illness*” OR “life-limiting condition” OR “life limiting condition*” OR “life-limiting disease” OR “life limiting disease*” OR “life-limiting syndrome” OR “life limiting syndrome” OR “life-limiting disability” OR “life limiting disabilit*” OR “ life threatening disease” OR “life-threatening disease*” OR “life threatening condition” OR “life-threatening condition” OR “end of life” OR “end-of-life” OR “terminal condition*” OR “terminal disease*” OR “terminal illness” OR “terminal disability” OR “incurable illness” OR “incurable condition*” OR “incurable disease*” OR “end-stage illness*” OR “end-stage condition*” OR “end-stage disease*” OR “terminal” OR “hospice” OR “end of life” OR “end-of-life” OR “last year of life” OR “stress, psychological” OR “basic needs” OR “world view” OR “affect” OR “experience*” OR “lived experience*” OR “view*” OR “thoughts” OR “feelings” OR “beliefs” OR “opinion*” OR “perceive*” OR “perspective” OR “needs” OR “priorit∗” OR “choice*” OR “preference” OR “job” OR “work*” OR “career” OR “decision*” OR “well-being” OR “wellbeing” OR “issue*” OR “matter*” OR “importan∗” OR “impact” OR “effect” OR “problem*” OR “challenge*” OR “barrier*” OR “obstacle*” OR “difficult*” OR “disadvantage*” OR “personal experience” OR “personal narrative” OR “attitude to illness” OR “psychological burnout” OR “money anxiety” OR “money stress” OR “money management” OR “money” OR “financ*” OR “financial anxiety” OR “financial distress” OR “financial hardship” OR “financial strain” OR “financial worr*” OR “financial burden” OR “financial toxicity” OR “economic anxiety” OR “economic burden” OR “economic impact” OR “economic hardship” OR “economic stress” OR “economic strain” OR “economic loss” OR “income anxiety” OR “income stress” OR “salary” OR “wages” OR “earnings” OR “financial support” OR “welfare benefits” OR “recourse to public funds” OR “public funds” OR “pay” OR “financial cost” OR “economic cost” OR “economic assessment” OR “economic evaluation” OR “economic implication*” OR “cost implication*” OR “indirect cost” OR “direct cost” OR “illness cost” OR “psychological wellbeing” OR “cope” OR “angst” OR “concern*” OR “suffer*” OR “uncertain*” OR “unease” OR “distress*” OR “defense mechanisms” OR “legalities” OR “rights” OR “law” OR “immigration law” OR “employment law” OR “employment rights” OR “labour law” OR “labor law” OR “legal services” OR “legal benefits” OR “authorities” OR “authority” OR “power dynamics” OR “distrust” OR “language barrier*” OR “foreign language” OR “different language” OR “second language” OR “other language” OR “non-native language” OR “non native language” OR “non-native english” OR “non native english” OR “partner” OR “informal care*” “relative*” OR “lay carer*” OR “informal support” OR “spousal support” OR “spousal caregiving” OR “family support” OR “access to healthcare” OR “healthcare access” OR “social behaviour” OR “behavioural symptoms” OR “cultural capital” OR “cultural inclusion” OR “cross-cultur*” OR “racial capital” OR “racial bias” OR “rac* discrimination” OR “rac* prejudi*” OR “cultural exclusion” OR “racial exclusion” OR “multicultural*” OR “social exclusion”
R: Qualitative research	“qualitative studies”[Mesh] OR “ethnographic research”[Mesh] OR “grounded theory”[Mesh] OR “observational methods”[Mesh] OR “focus groups”[Mesh] OR “interviews”[Mesh] OR “multimethod studies”[Mesh] OR “action research”[Mesh] OR “discourse analysis”[Mesh] OR “case studies”[Mesh] OR “narrative analysis” OR “photovoice” OR “photo-elicitation” OR “photo elicitation” OR “qualitative” OR “qualitative research” OR “ethnograph∗” OR “phenomenological research” OR “phenomenology” OR “phenomenol∗” OR “hermeneutic∗” OR “observation∗” OR “mixed method∗” OR “mixed-method∗” OR “multimethod∗” OR “multi-method∗” OR “questionnaires” OR “surveys” OR “participatory action research” OR “case study” OR “I-Poem” or “I Poem” OR “purposive sampl*” OR “cluster sampl*” OR “heuristic*” OR “semiotic*” OR “narrative*” OR “content analysis” OR “thematic analysis” OR “constant comparative method” OR “field stud*” OR “theoretical sampl*” OR “ethnomethodology*”

MEDLINE (via PubMed), CINAHL (via EBSCO), PsycINFO (via EBSCO), SocIndex (via EBSCO) and Web of Science databases were searched. A pilot/scoping search was conducted in CINAHL to refine the search strategy. Search limiters, Boolean operators, and wildcards/truncation (*$?), where necessary, were used to balance the search’s specificity and sensitivity [46].

### Procedure

A comprehensive search of each of the databases was conducted from 01/01/2000, with searches performed between 28/08/23 and 30/08/23. Citations were imported into Rayyan Computing Research Institute (QCRI) Software [47] for suitability screening. Three blinded reviewers (TS, LF, and JA) independently screened titles and abstracts against the inclusion criteria to mitigate the risk of bias [48] and removed ineligible studies. Full texts of remaining articles were screened against the inclusion criteria by the same three reviewers, and any disagreements were resolved in consensus meetings between all three.

### Risk of bias

The critical appraisal skills programme (CASP) qualitative checklist was used to assess quality and risk of bias. Although not as sensitive to theoretical, interpretative, and evaluative validity, or the intrinsic methodological quality of some appraisal tools [49], the CASP checklist is the most used appraisal tool for evidence synthesis [50, 51]. Bias was rated in the first 9 questions as being low, medium, or high, following which they were converted into ordinal scores (low = 1, medium = 2, high = 3); the lowest scoring studies were regarded as the highest quality, having the least risk of bias.

Most studies were appraised to be of low bias risk, except for two studies which were deemed to be of moderate risk [52, 53], and one study assessed as having a high risk of bias [54], which may reflect study reporting rather than study conduct/quality [55].

The exclusion of studies demonstrating any weakness in methodology is a contested practice [56]; data synthesis may be weakened if studies are excluded since this may reflect reporting rather than research conduct. Consequently, no studies were excluded based on their quality appraisal [57].

### Data extraction and synthesis

Qualitative evidence was deemed to be the textual data within the abstract, findings, or discussion sections of the included studies [37, 58]. All data were extracted by two reviewers (TS and LF) and imported into NVivo 14 for more detailed coding, which was performed by two reviewers (TS and JA), and which facilitated the identification of emerging themes and trends through a process of systematic retrieval, visualisation, and recording of more detailed memos [59].

Themes were refined, named, and reviewed through multiple recursive cycles [37]. Categories and second order analysis were developed by two reviewers (TS, and JA), who, with the third reviewer (LF), reached full agreement on the final descriptive and analytical themes of the study.

The synthesis was appraised using GRADE-CERQual, to assess the confidence of the findings being a representation of the phenomenon of interest (i.e. first generation migrant experiences of palliative care) [60].

## Findings

**Fig. 1:**
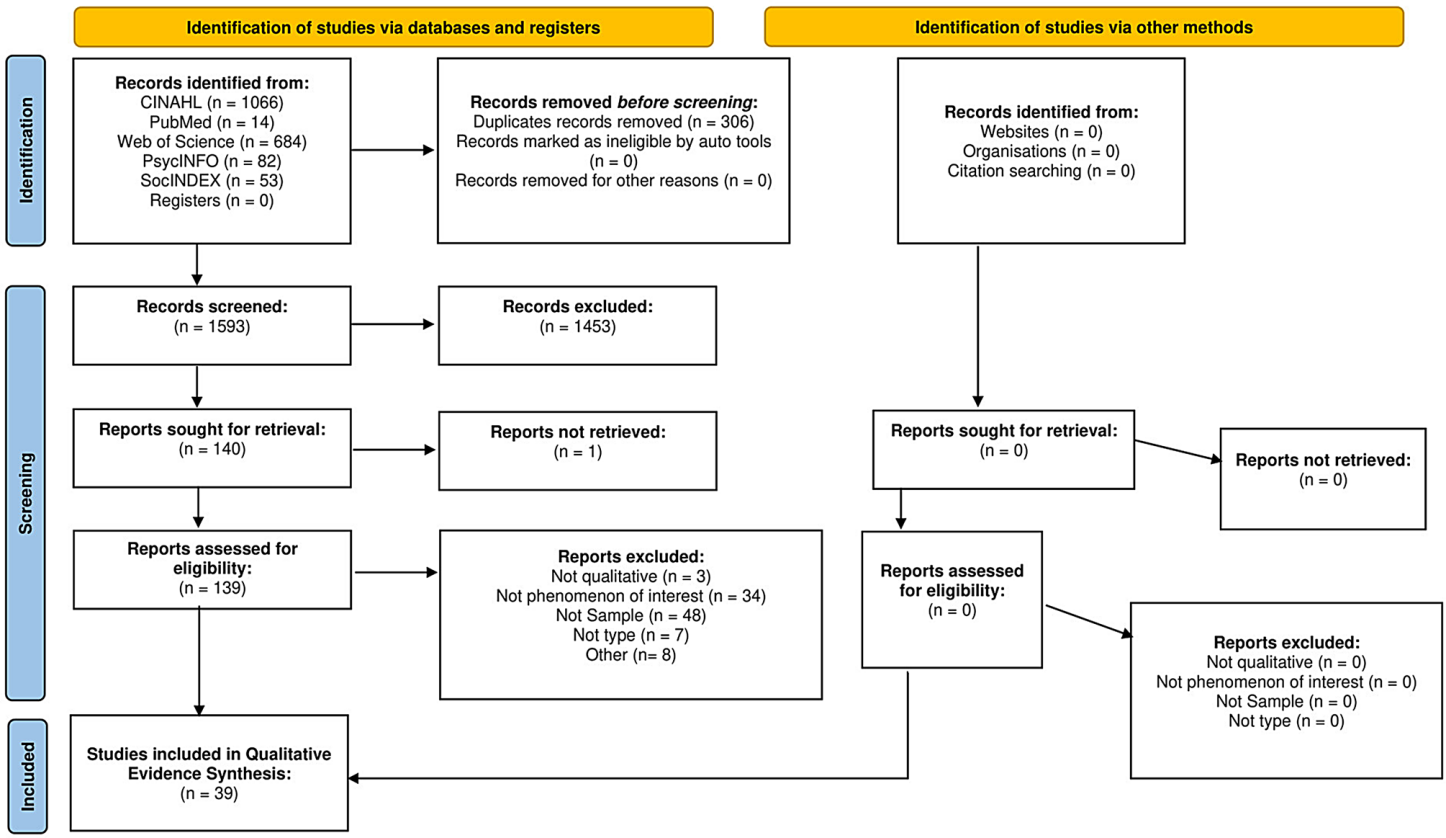
PRISMA flow diagram: study identification, screening and selection

Database searches identified a total of 1593 records following the removal of duplicates. From these 1593 records, 1453 were excluded, leaving 140 for retrieval. Of the 140 records, 139 were retrieved (with the final publication being a PhD thesis which was not accessible). From these records 100 were excluded, leaving 39 for inclusion in the qualitative evidence synthesis. Primary reasons for exclusion were because they were not from the sample of interest, did not explore the phenomenon of interest, or were not qualitative. Figure 1 highlights the study selection process [39].

Included studies were conducted between 2001 and 2023, all were qualitative, except for two studies which were mixed methods [61, 62] from which we extracted qualitative data. One study was a systematic review [63]. Studies were conducted across eleven countries from four continents, primarily North America and Europe; the characteristics of which are highlighted in Table 2. Despite this heterogeneity, there were some compelling similarities across studies and locations. Table 3 outlines the assessment of confidence in the review findings.

**Table 2:** Study characteristics and critical appraisal

Author	Year	Location	Design	Sample size	Mean age [range]	Participant	CASP risk of bias rating
Angelo	2014	New Zealand	Qualitative	6 [*n* = 4 = 1st gen.]	51.8 [38–67]	Patient	Low
Beltran	2022	USA	Qualitative	13 [+ 10 proxies]	82 [patients] 54 [proxies]	Both	Low
Bray	2007	New Zealand	Qualitative	4	Unknown	Family	Low
Bray	2018	New Zealand	Qualitative	10	60.7 [42–88]	Patient	Low
Carrion	2007	USA	Mixed method	10 hospice carers [*n* = 8 family / *n* = 2 professionals] 10 non-hospice carers [*n* = 4 family / *n* = 6 professionals] 10 physicians	Unknown	Both	Low
Carrion	2010	USA	Qualitative	20 [*n* = 10 hospice users / *n* = 10 non-hospice users]	Unknown	Both	Low
Carrion	2013	USA	Qualitative	20 [*n* = 13 carers; *n* = 7 paid carers]	54 [20–75 in family carer group]	Family	Low
Chiang	2015	Australia	Qualitative	22	54 [37–81]	Patient	Low
Chui	2005	Australia	Qualitative	11	48.7 [36–58]	Patient	Low
de Voogd	2020	Holland	Qualitative	23 patients 21 relatives	[45–55] - [86–95]	Both	Low
de Voogd	2021	Holland	Qualitative	20	[36–40] - [70–75]	Family	Low
del Gaudio	2020	USA	Qualitative	1 family [*n* = 4 = members] 4 families [not meeting SR criteria]	21.75	Both	Moderate
Diver	2003	UK	Qualitative	4	Unknown	Patient	Low
Dosani	2020	Canada	Qualitative	34	Unknown	Patient	Low
Gaveras	2014	UK	Qualitative	20 patients 6 carers 9 professionals	Unknown	Both	Low
Glasdam	2023	Denmark	Qualitative	2 cases	Unknown	Patient	Low
Glaser	2020	USA	Qualitative	13 patients 12 carers 10 professionals	56 [patients]	Both	Low
Guo	2021	Jordan	Qualitative	29 patients 7 carers	55 [26–75] patient 42 [31–55] carers	Both	Low
Heidenreich	2014	Australia	Qualitative	5 female carers	Unknown [*n* = 1 age 50–55; *n* = 4 aged 56–65]	Family	Low
Helsel	2020	USA	Qualitative	15 carers 5 spiritual carers/funeral officiant	41 [all participants]	Family	Low
Hong	2012	Singapore	Qualitative	15	73 [66–83]	Patient	Low
Kim	2009	USA	Qualitative	8	67 [48–84]	Family	Low
Koffman	2001	UK	Mixed method	50 Black Caribbean migrants 50 White non-migrants [comparison]	[18–44]-85+ [all participants]	Family	Low
Kreling	2010	USA	Qualitative	15 Latino migrants 15 White non-Latinos [comparison]	55.1 [38–88]	Family	Low
Kristiansen	2016	Denmark	Qualitative	1	‘Late 50s’	Family	Moderate
Kwok	2020	UK/USA/ Australia/Canada	Qualitative systematic review	7 studies which included: 82 patients 23 carers 21 professionals	Unknown	Both	Low
Lin	2008	USA	Qualitative	12	[37–70]	Patient	Low
Lou	2021	Canada	Qualitative	31 [*n* = 3 focus groups: group 1 *n* = 5; group 2 *n* = 12; group 3 *n* = 14]	58	Family	Low
Migala	2020	Germany	Qualitative	29	Unknown	Both	Low
Nedjat-Haiem	2013	USA	Qualitative	24	52 [35–79]	Patient	High
Neilsen	2013	Canada	Qualitative	4 patients 11 key informants [*n* = 5 family; *n* = 6 other]	50–80 [patients]	Both	Low
Neilsen	2015	Canada	Qualitative	4 patients 11 key informants [*n* = 5 family; *n* = 6 other]	50–80 [patients]	Both	Low
Paal	2017	Germany	Qualitative	18 migrants 19 native Germans [comparison]	70 [32–95]	Patient	Low
Saachi	2021	Italy	Qualitative	27 [*n* = 5 family]	48.3 [all participants] [31–68 all participants]	Family	Low
Seymour	2007	UK	Qualitative	46 Chinese migrants 32 White participants [comparison]	< 55–85+ [both groups]	Family	Low
Shanmugasundaram	2009	Australia	Qualitative	6	[47–68]	Family	Low
Shanmugasundaram	2015	Australia	Qualitative	6	[47–68]	Family	Low
Venkatasalu	2017	UK	Qualitative	55	Unknown	Family	Low
Weerasinghe	2016	Canada	Qualitative	7	Unknown	Family	Low

**Table 3:** GRADECerqual

Summary of review finding	CERQual assessment of confidence	Explanation of assessment (methodological limitations, coherence, adequacy of data, relevance)	Studies contributing to the review findings
Access to healthcare: compromised due to financial costs, language barriers, culturally unresponsive care	High confidence	Very minor concerns regarding methodological limitations (primarily associated with reflexivity), coherence and adequacy, and minor concerns regarding relevance which at times focused on broad experiences of receiving palliative care rather than focused on the impact of being a migrant in that context.	Beltran et al. (2022); Bray and Goodyear-Smith (2007); Chiang et al. (2015); Chui et al. (2005); Diver (2003); Glasdam (2023); Glaser et al. (2020); Guo (2021); Lin (2008); Heidenreich et al. (2014); Helsel et al. (2020); Hong et al. (2012); Kim (2009); Kreling et al. (2010); Migala and Flick (2019); Nielsen et al. (2015); Nielsen et al. (2013); Shanmugasundaram and O’Connor (2009).
Burden of financial precarity: working while ill, costs associated with dying/death.	High confidence	Very minor concerns regarding methodological limitations, coherence and adequacy, and no concerns regarding relevance of financial circumstances being linked with immigration status and ill-health.	Beltran et al. (2022); Glasdam (2023); Glaser et al. (2020); Guo (2021); Heidenreich et al. (2014); Hong et al. (2012); Lin (2008); Nedjat-Haiem et al. (2013); Shanmugasundaram (2015).
Receiving and providing care and treatment: culturally appropriate and insensitive care, comparisons with country of origin and family care.	High confidence	Very minor concerns regarding methodological limitations, coherence and no concerns regarding adequacy or relevance of the impact of migration status impacting care receipt.	Angelo and Wilson (2014); Beltran et al. (2022); Bray and Goodyear (2007); Carrion (2007); Carrion and Nedjat-Haiem (2013); Chiang et al. (2015); de Voogd et al. (2020); de Voogd et al. (2021); Del Gaudio et al. (2013); Diver (2003); Gaveras et al. (2014) Guo (2021); Helsel et al. (2020); Hong et al. (2012);Kim (2009); Koffman et al. (2001); Kreling et al. (2010); Kristiansen et al. (2016); Kwok et al. (2020), Lin (2008); Linn (2000); Nielson et al. (2015); Paal and Bukki (2017); Sacchi et al. (2021); Seymour et al. (2007); Shanmugasundaram and O’Connor (2009); Shanmugasundaram (2015), Venkatasalu (2017); Weerasinghe and Maddalena (2016).

Excerpts of data are included below; we include participant characteristics where these were provided by the original papers to contextualise the quotations. Three themes were developed from the data: (i) access to healthcare; (ii) financial precarity, (iii) receiving and providing care.

### Access to healthcare

Access to healthcare included four sub-themes: financial barriers, language barriers, lack of care provider understanding, and services not being inclusive.

Many first-generation migrants reported inability, or fear of the inability, to pay for healthcare, treatment, and medications, in those countries where healthcare was not free at point of access. Since the data corpus drew on multiple countries where there was no free healthcare, this was a serious concern for many [64–67]. For some, lack of finances resulted in their inability to access essential medical supplies or in the premature discontinuation of their treatment for terminal illness when they could no longer afford to pay for it. The following extract comes from a study of refugees in Jordan where considerable money had been needed to finance treatments for many of their respondents:

I have 6 daughters and 2 sons, with me and my husband. We are 10, and I have been receiving treatment for 4 years at my own expense over 12K, half of them are debts…This cycle is probably the last one I am gonna take because I can’t afford any more. I can barely get my children bread. (Patient aged 45, female, Syrian, Breast Cancer IV - PAL0037). [66 p923]

Conversely, those with health insurance were grateful that this gave them confidence and ability to access the necessary healthcare and treatments related to their terminal illness [68]. Paradoxically, for some people with insurance, uncertainty and fear of unknown charges served as a barrier to seeking essential healthcare services, one Latino decision-making proxy of a hospice user said:

I hope her insurance covers all of this … that later they don’t come with a list [saying] that I owe x amount of money because, from where? I have that worry. [64 p155]

When healthcare was accessed, this helped resolve social isolation that they felt as migrants [69–71]; although for others, inability to speak the language of the country they migrated to only served to reinforce experiences and feelings of exclusion [71].

Language and communication issues were predictably widely reported as problematic for migrants, in terms of access to and understanding of healthcare and the healthcare system. Migrants often relied on relatives to translate, including discussions of prognosis, the types and availability of services, care coordination, requests for more assistance, and communicating their needs and wants [64, 71–73].

Even when migrants perceived healthcare providers as knowledgeable and able to understand them, they often still felt alienated [71, 73–75]. Some participants appeared to create a bridge from their adopted country to their country of origin by creating and/or maintaining social connections [73, 75, 76]. This was observed specifically in terms of their drive to access traditional treatment modalities from their homeland and/or engage the services of healthcare providers from their own culture, as an adjunct or alternative to treatments in their adopted country [73, 75, 76]. For some, maintaining social connections with their place of birth was also partly driven by not being able to sufficiently communicate in the language of their adopted country:

‘Because most of my friends in China are doctors, I can call them and ask for advice’ (Sue, translated). […] Despite her limited English, she was creative in accessing relevant information, and also tapped into pre-existing transnational linkages. [77 p653]

For some migrants, access to appropriate healthcare treatments in their country of origin were scarce [78], or impossible due to the instability of war [66]. Yet, this notion of wanting to have a foot in both countries is an interpretation by the authors, rather than expressly articulated by participants. Consequently, the liminal space of being in both, but neither country fully, is perhaps an artefact of circumstance (being unable to access appropriate services in the country of residence) rather than choice. Gaining support from friends is a resourceful approach, drawn on in the absence of suitable services.

The literature tended to cast migrants as lacking understanding and awareness of healthcare and palliative care services [64, 75, 79, 80]. Indeed, there is a noticeable lack of reflexivity in published papers regarding the ways that comprehension and navigation of healthcare is helped or hindered by providers. Although some people made efforts to network and engage with specialist cancer organizations [76], other papers reported that migrants found the topic of terminal illness too distressing, and therefore limited their exposure to information. The following quotation was presented as a Latino’s view in contrast to white non-Latinos desiring detailed information:

They gave me a pamphlet of what to expect. It explained all the steps my mother would go through until the day she would die. I did not want to read it. It was a plan or a guide I did not want to know or wanted to do. They told me I had to read it to be prepared. Even though it was practical advice about how to handle “the end” I felt it was very drastic. [80 p6]

Overall, service providers were not blamed for their lack of inclusive practices; the onus was placed on migrants to source information and access healthcare. Yet resisting pamphlets, for example, can be cast as a reasonable response to a dehumanised and an unsophisticated approach to communicating about end of life and dying. Migrants are not expressly refusing care or support, but questioning the appropriateness, accessibility, and framing of services. Consequently, within the evidence to date, papers do not call-out potential systemic culturally insensitive practices within the healthcare system. For example, papers focused on individual lack of understanding of services, which situates the problem as a deficit in the migrant, rather than the system [64, 75, 79, 80].

### The burden of financial precarity

The burden of financial precarity included four sub-themes: the cost of care, employment, the cost of dying, and family support. This builds on the prior theme by reporting the consequences of financial strains beyond accessing health systems.

The data demonstrate the considerable burden of financial precarity experienced by migrants and their families [64–68, 70, 75, 81]. Less financial stress was experienced by people with health insurance or healthcare provided by the State, even with minimal or no income, as this meant being largely worry-free about receiving treatments or attending follow-up appointments [68]. Guo’s [66] study of terminally ill migrants in Jordan, where healthcare is not free at point of delivery, summarises: “*Poverty in the face of expensive treatment caused greater emotional pain” (p922).*

The overwhelming sentiment of many was that ‘[t]he biggest concern is always the economic situation’ [65 p1291]. Thus, despite substantial symptom burden, for many people giving up work was not an option because of the threat that financial precarity posed to family in their current country and country of origin [64, 65]. The following quotation includes experiences from two different speakers:

There are times I get up…I say, ‘God, give me strength’…If I had money, I’d stay in bed…but I have to support my nine-year-old son… […] “My wish for the future is that God keeps me positive and to be able to continue working…I have a brother who is 85 years old. I send him a little money to help with food, a bit of medicine…in our homeland poverty is worse than ever…if I don’t send them that money, how will they live? [65 p1291]

The financial burden experienced when forced to give up work due to ill health caused stress for some and impacted their ability to support themselves and pay for housing, medicines, and medical supplies [66]. This caused embarrassment due to reliance on others for financial help.

Financial and immigration constraints also precluded visiting other dying relatives and attending their funerals, and which reduced their own experiences of confronting death [64]. Financial constraints also prevented people from being able to return to die and/or be buried in their country of origin [65].

The weight of facing the expense associated with dying was especially concerning [70, 82]. The cost of dying became the main focus for some, as the following Latina in the USA with advanced cancer explained:

If one begins talking about death, one doesn’t talk like right now about being prepared. You talk about debt that you leave. You talk about buying a little plot where you’ll be buried. That’s what one thinks when one thinks about death. So, that’s why I prefer not to talk about that because it’s very expensive to die. [54 p171-172]

Additional burden was experienced by those who were once the primary breadwinners, when no longer able to work and financially provide for their families [68, 70]. The following quotation from an Australian study demonstrates considerable distress:

My husband was the only earning member in the family. Now he is suffering with cancer and admitted to the hospital. I do not know anything about the outside world. I have no money for the treatment I stress about finance. (Caregiver 5, female, aged 47, wife of patient). [67 p540]

Financial stress was one of many impacting the lives and deaths of first-generation migrants. The following authors’ comment summarises data from US-resident Chinese migrants on their experiences of living with metastatic cancer:

Worry about one’s family, worry about becoming a burden, worry about financial stress, uncertainty about the future, and guilt about not having paid attention to one’s health needs before being diagnosed with cancer also contributed to their psychological and spiritual suffering. [68 p253]

The financial burden suffused their experience. It ran from initial concern about paying for treatment and care, through to working-while-ill, and into the era after their death, when they worried about how their funeral costs would be covered and the family would manage after they died.

### Receiving and providing care

Receiving and providing care included three subthemes: culturally insensitive care from services, comparisons with country of origin, and family care.

Both culturally informed/congruent care and insensitive practices were reported; however, culturally insensitive practices were more dominant [61, 69, 78, 79, 83–85]. Some structural and staff educational barriers to care provision were reported, for example, insensitivity to the need for fasting, which was perceived as clashing with treatment regimens, no provision of culture-specific diet, and the lack of designated quiet space for prayer [79]. Families cited the insensitivity of staff in terms of being patronising and not acknowledging patient ‘norms and values’ [85], making people uncomfortable when praying [61], not informing them promptly that their relative was in the final stage of their life, which prevented them from performing specific end-of-life rituals [79, 84].

When healthcare providers were culturally attuned [62] some migrants shared this experience within their communities, which reinforced continuation of such practices amongst healthcare and other professional service providers [86]. Consequently, when migrants felt welcomed and cared for, this acted as a virtuous cycle encouraging others from marginalised groups to access services.

In some studies, people with terminal illness and families reported satisfaction with the standard of care received [87], and that this was better than was available/could be accessed in their country of birth [86, 88]. Satisfaction was linked to perceived competence which instilled a level of trust [82], and where professional expertise and good interpersonal skills helped to foster confidence in care quality. One master’s educated Latina respondent said of her father’s doctor:

He just really knew his stuff […] I did my homework and oh my god. He [doctor] had more than 10 years’ experience. Robot surgeries, all these specializations. [64 p156]

The interplay between staff care and attitudes and the impact on patient and carers ran through this theme. When families were determined to provide care to their relatives themselves, this improved their self-image [74] and appeared to bolster the dignity of those for whom they were caring [66, 68, 89]. However, when families and patients felt their views were disregarded, they felt devalued [89], with some implying that this was due to religious or cultural differences. The following quote from a daughter of a person with paralysis from a cerebral haemorrhage said:

I really had the feeling she wasn’t suffering [from pain]. [.] But the physician kept constantly saying, “Morphine, morphine, and stop the medication. Stop the tube feeding.” I thought, “Don’t you have anything else to say? We’ve already said ‘no’ several times. What are you doing in an Islamic unit?… Don’t you take me seriously as a legally designated representative? We didn’t just pick something. We really gave it thought together, as a family. [89 p7-8]

Migrants felt that they needed to contain their feelings about illness progression, by not complaining, initiative-taking, and playing an active role, including in the latter stages of terminal illness [89, 90]. Notions of *being a good patient* were also apparent [53, 63, 75]. For some, being proactive, such as actively joining or being associated with cancer support groups, was discouraged by friends and family members [72]. In some circumstances patients were avoided by friends and family, which was reportedly driven by cultural beliefs and the fear of acquiring misfortune by association [91]. Additionally, when decisions were made for care to be provided outside of the family home, othering and isolation were experienced by some families, which they found upsetting:

We cannot be part of the community anymore. The gossip, it drives me nuts (crying tone). They say: ‘He has dumped his wife there and he is just gallivanting on his own.’ They only see the appearance, nobody knows the inner. *Interviewer: Do you have fears regarding her dignity in the last phase of life?* Respondent: They come from outside, impair her feeling of dignity and also mine. I relinquished from the mosque, I do my prayers here. (Turkish relative, #28) [85 p1389].

The data indicated that despite being fully aware of their relative’s prognosis, directness of communication in terms of dying and death was experienced by some relatives as being brutal, and appeared to be linked to different expectations:

As a Latino the fact that they tell you straightforward that your husband is dying…the doctor tells you ‘he is at the end of his life’; it sounds a little cruel…I knew there was no cure for him […] [that it] was palliative only. However, still it made me angry when the doctor told me he is dying. [80 p6]

Placing their health and needs last, some family members viewed caring for the person who was dying as a duty and their final opportunity to give back to them before death [79], whilst others took strides to provide support which was unobtrusive [73]. Respondents expressed sadness at not having more family close by [67, 92]. Some developed strategies by not discussing terminal illness with their dying relatives [75], whilst others avoided engaging with outside agencies and hid the terminal diagnosis from professionals [63] and younger family members [52]. Withholding the prognosis from the patient meant recruiting healthcare providers into this secret-keeping. The following Spanish speaker *born in the Dominican Republic* said:

I told her [Spanish-speaking counselor], ‘’Listen, I didn’t let her know the extent of what is happening.I didn’t want her to start freaking out.‘’ She said, ‘’Don’t you think she needs to know…so that she could get ready? At what point will you tell her?‘’ [.]’ She told the counselor to ‘’respect what I’m saying in my house because I know what is best for her, you don’t.‘’ [93 p186-187].

## Discussion

Health services and treatments were not experienced as easily accessible by first generation migrants. Lack of support for translation/interpreting fed into language barriers, and constrained social support was also evident. Financial precarity ran as a thread through the data, with participants needing to work while unwell, and being unable to return to their country of origin for their own death or to bear witness to the deaths of relatives. First generation migrants experienced caregiving through the lens of difference; maintaining autonomy in the country they would die in, intersected with cultural practices and expectations such as not sharing the prognosis, and mis-matched ideas regarding quality of care provided.

The findings are an amplified variant of data from non-migrant populations, including the considerable financial strain on families, especially where healthcare is very costly, and imposes the need to make decisions about caregiving, resting, or working [94]. For first generation migrants, these burdens are disproportionally felt. Lack of finances means that for some people, stopping work is not an option despite having a terminal illness. Social capital, the connections between people that can provide support, resources and opportunities [95], is likely to be much weaker for people without the depth or breadth of social networks in their new country. In practice, this means first generation migrants are more isolated and vulnerable [96]. Social capital has been recognised as correlated with migrant quality of life [97]; impoverished social networks are an independent risk factor for poor health [98], and hence it is not a big leap to assume it would also be associated with worse quality of dying and death. Without sufficient financial and social support resources, first generation migrants are at risk of being unable to focus on, and plan for, their end of life.

Receiving and providing care evokes the ethics of care principles [99], and an obligation to address causes of harm. Care providers have a moral duty toward attentiveness, responsibility, competence, responsiveness, and trust, yet appear to fall short of those principles for migrants with a terminal illness. Addressing structural barriers must occur prior to diagnosis of a terminal illness, as well as into the bereavement phase.

The burgeoning focus on equity in palliative care needs to identify and dismantle structures which uphold and perpetuate inequality. Palliative care policies may cite marginalised groups such as people from minoritised ethnic groups, yet often this is not accompanied by specific recommendations or requirements to address first generation migrants who face multiple layers of disadvantage. Accounts of religion, belief and spirituality being discounted or not respected were woven through the papers, reflecting recognised deficits in service organisation and delivery [100, 101].

While the financial impact of caring is recognised [102], there has yet to be detailed focus on migrants facing terminal illness, and the considerable repercussions felt by people subject to immigration controls on their income and rights. Service commissioning and strategies to widen access must be cognisant of the complex constellation of circumstances in which migrants live and die. Palliative care has not, in real terms, become a human right [103, 104], though there are legislative approaches being developed in some countries [105]. Diasporic dying is not a new phenomenon [31], yet services and policies fail to meet people’s needs. Transnationalism and intersectionality are still not well understood in the context of dying migrants [31], despite recognition that there are important nuances and differences. Research focused on migrants is important in ensuring a robust evidence base from which to change policy and practice [14] given the unique circumstances they live and die in.

There remains a considerable knowledge gap in understanding specific migrant experiences, for example, refugees and people seeking asylum. Theorising around economies of kinship, that is, the role that transnational families play in unpaid care [106] could be extended to examine the lives and deaths of terminally ill migrants. Relational theories may also be useful to produce insights into transnational communication and managing co-presence in caring for dying diaspora [107, 108]. The financial burdens of dying and post-death care requirements (such as funerals) are likely to impact individual and family experiences in the home-land and the host-land [109], in ways that we do not fully yet understand.

The paucity of positive experiences signals a need to change practice and policy. One step toward this would be adopting a co-design approach to health care policies with migrant communities. Such co-design would offer opportunities to attend to the heterogeneity of collectivist and individualist approaches to care, enhance cultural appropriate services, increase uptake of services, and attend to the different life and death experiences of migrants.

### Limitations of the review

Papers included in this review report studies conducted in various countries with participants of differing ethnicities; the studies’ samples therefore included considerable variation in terms of people’s culture, customs, language and experiences. This reflects the heterogeneity within respondents labelled as ‘migrants’ [110]. The possibility for cultural bias or omissions in interpretation is acknowledged, in terms of excluding non-English publications, which may limit transferability because some culturally specific issues may not have been captured [111]. Further, the range of countries meant heterogeneous healthcare systems, including some with free palliative care and others where a co-pay or full payment is required, which directly impact the accessibility and affordability of care.

As with all systematic reviews, the search may have not identified all relevant studies due to articles/books not being indexed by databases thoroughly, for example some of Gunaratnam’s body of work [31]. Nevertheless, since recurring themes and ideas were repeated across the included sources, and replicate (if not as eloquently) Gunaratnam’s work, we are confident that the qualitative evidence synthesis represents the experiences of first-generation migrants living with a terminal illness.

The findings represent a problematising approach to understanding migrant experience. This deficit focus reflects the data from the papers included in the review [63, 71, 79, 80], though we recognise that migrants are also highly likely to bring resilience and strengths, including and beyond the impact of stereotypes such as faith on coping [53, 68] and support of co-resident family [84, 112].

## Conclusions

We do not want this review to reify a homogenous view of migrants but wish to highlight the specificities that holding this legal status has on access to healthcare, use of healthcare, and the perilous impact that terminal illness has on financial security. Migrants with terminal illness occupy a liminal space; they are both *in the new country*,* and in the old; they are both known and knowledgeable and yet othered*. They access healthcare, yet are keenly aware of their difference. They work while ill, yet are too ill to work.

The cost of accessing care, medicines and support alongside people’s fragile immigration status means that uncomfortable choices may need to be taken, regarding whether to earn money or rest. An entire family’s immigration status may depend on the employment of the person affected by illness. Those affected therefore face the dilemma of continuing to work while unwell, ceasing work, or relatives undertaking undocumented work. The experiences of migrants without settled status is an important subgroup where there is limited understanding of how they can live and die well with a terminal illness.

Future research should be conducted with an intersectional approach and where nuance and description of experience can be more precisely described. In doing so, policymakers and healthcare providers can make informed decisions to develop appropriate and accessible services which better meets their needs.

## Data Availability

All articles are published and interested parties should use the citation to gain access to raw data.

## Appendix 1

**Definitions**

Destitution: extreme poverty.

First generation migrant: foreign born person who moves to another country.

Forced migrants: people who have left their home country due to factors outside their control such as war/persecution, including refugees and people seeking asylum.

Undocumented migrant: someone who has moved to another country but does not have the relevant authorisation (such as a valid visa).

Migrant: a person who moves from their country of birth to another country.

Transnational people: a person who has lived in more than one country.
